# A scientometric analysis of research on the role of NMDA receptor in the treatment of depression

**DOI:** 10.3389/fphar.2024.1394730

**Published:** 2024-06-21

**Authors:** Xulin Chen, Xian Wang, Caijuan Li, Yao Zhang, Shanwu Feng, Shiqin Xu

**Affiliations:** Department of Anesthesiology, Women’s Hospital of Nanjing Medical University, Nanjing Women and Children’s Healthcare Hospital, Nanjing, China

**Keywords:** ketamine, NMDA receptor, depression, scientometrics, bibliometrics, evidence synthesis, research trends

## Abstract

**Background:**

There have been numerous studies on NMDA receptors as therapeutic targets for depression. However, so far, there has been no comprehensive scientometric analysis of this field. Thus, we conducted a scientometric analysis with the aim of better elucidating the research hotspots and future trends in this field.

**Methods:**

Publications on NMDAR in Depression between 2004 and 2023 were retrieved from the Web of Science Core Collection (WoSCC) database. Then, VOSviewer, CiteSpace, Scimago Graphica, and R-bibliometrix—were used for the scientometric analysis and visualization.

**Results:**

5,092 qualified documents were identified to scientometric analysis. In the past 20 years, there has been an upward trend in the number of annual publications. The United States led the world in terms of international collaborations, publications, and citations. 15 main clusters were identified from the co-cited references analysis with notable modularity (Q-value = 0.7628) and silhouette scores (S-value = 0.9171). According to the keyword and co-cited references analysis, treatment-resistant depression ketamine (an NMDAR antagonist), oxidative stress, synaptic plasticity, neuroplasticity related downstream factors like brain-derived neurotrophic factor were the research hotspots in recent years.

**Conclusion:**

As the first scientometric analysis of NMDAR in Depression, this study shed light on the development, trends, and hotspots of research about NMDAR in Depression worldwide. The application and potential mechanisms of ketamine in the treatment of major depressive disorder (MDD) are still a hot research topic at present. However, the side effects of NMDAR antagonist like ketamine have prompted research on new rapid acting antidepressants.

## 1 Introduction

Depression is a psychiatric disorder which affects emotions, behavior, and general health. People suffering from depression may also experience loss of appetite, insomnia, and difficulty in concentrating (Malhi and Mann, 2018). Furthermore, people with major depression are often accompanied by suicidal ideation. With a global incidence of 4.4% (Marwaha et al., 2023), depression affects over 300 million individuals worldwide and is a prevalent and recurring (Friedrich, 2017). Depression has emerged as a major global mental health concern and the primary cause of disability connected to mental health globally (Herrman et al., 2019). A significant fraction of people with depression do not react to current treatments, in spite of the multiple antidepressants and therapeutic strategies available. The current first-line antidepressants, like SSRIs (selective serotonin reuptake inhibitors) and serotonin-norepinephrine reuptake inhibitors (SNRIs), require weeks, if not months, to generate a response. The underlying mechanisms of these antidepressants are based on the monoamine system. Nevertheless, long-term usage of these medications has been linked to cardiotoxicity, trouble sleeping, and sexual dysfunction as side effects (Rush et al., 2006). Given this, it is now generally acknowledged that to enhance patient prognosis, drug discovery needs to expand outside the monoamine system. Particularly, the glutamate system has become a dynamic research field (Murrough et al., 2017; Marwaha et al., 2023).

Along with other ionotropic receptors (AMPA receptors and kainate receptors) and G-protein coupled receptors, N-methyl-D-aspartate receptors (NMDARs) are a class of cation-selective ligand-gated ion channels that mediate glutamatergic synaptic transmission all over the central nervous system (Traynelis et al., 2010; Hansen et al., 2021). In general, NMDARs are heteromeric tetramers containing two glycine-binding GluN1 subunits and two identical glutamate-binding GluN2 subunits (GluN2A- GluN2D) (Hansen et al., 2018). Nevertheless, NMDARs can also be composed of two GluN1 and two glycine-binding GluN3A/GluN3B subunits (Paoletti et al., 2013; Hansen et al., 2021). The molecular structure of each GluN subunit is similar and consists of four semi-autonomous domains: a transmembrane domain (TMD) which contains 4 transmembrane segments (M1-M4) and the M2 segment forms a ion channel, the ligand binding domain (LBD or the agonist binding domain, ABD), an intracellular carboxyl tail domain (CTD), and the aminoterminal extracellular domain (NTD) (Hansen et al., 2017; Lu et al., 2017). NMDARs are essential for neurodevelopment, synaptic plasticity, sensory/motor integration, learning and memory (Gécz, 2010; Paoletti et al., 2013; Burnashev and Szepetowski, 2015; Hansen et al., 2021). Glutamate is the primary excitatory neurotransmitter in the central nervous system, and NMDARs are essential for synaptic plasticity. As a result, dysregulation in NMDAR function is associated with a variety of neuropsychiatric disorders, including schizophrenia, addiction, anxiety, and depression (Murrough et al., 2017; Chen et al., 2018; Yang et al., 2018; Daneshparvar et al., 2019; Uno and Coyle, 2019; Salimando et al., 2020; Shin et al., 2020; Ma et al., 2023). Considering the physiologic role and pathological impact in the CNS, NMDARs are potential targets for developing therapeutic agents for neuropsychiatric disorders.

It is commonly established that NMDAR and depression have a strong connection (Petrie et al., 2000; Gerhard et al., 2016; Amidfar et al., 2019). Patients with MDD have been shown to have dysfunctional glutamatergic neurotransmission (Skolnick et al., 2009; Musazzi et al., 2013; Kim and Na, 2016), and there have been reports of NMDAR complex dysfunctions in the frontal cortex of suicide victims, including decreased glutamate recognition site and glycine-mediated allosteric modulation of this site (Nowak et al., 1995; Karolewicz et al., 2005). Moreover, long-term antidepressant drug therapy causes adaptive modifications in the NMDA receptor’s binding properties (Nowak et al., 1993; Paul et al., 1994). Antidepressants may modify the subunit makeup of NMDARs as well as the region-specific expression of NMDARs, according to a few animal experiments (Nowak et al., 1993; Nowak et al., 1996; Skolnick et al., 1996; Boyer et al., 1998; Feyissa et al., 2009). Therefore, these adaptive changes in NMDAR may be the ultimate common pathway in the mechanism of action of antidepressants. In addition, numerous studies have revealed that NMDAR antagonists have rapid and sustained antidepressant effects (Berman et al., 2000; Zarate et al., 2006; Krystal et al., 2019; Phillips et al., 2019). In summary, NMDAR is an important future therapeutic target for depression, but our understanding of the relationship between NMDAR and depression remains vague.

Numerous reviews in recent years have offered explanations of the role of NMDAR in depression (Amidfar et al., 2019; Hanson et al., 2023; Zhou and Duan, 2023). It is important to use novel methods to uncover underlying connections and trends given the abundance of research in this field. Recently, a novel method known as “research weaving” was presented (Nakagawa et al., 2019). It combines bibliometrics with systematic mapping to facilitate the synthesis of research on both influence and evidence. Systematic mapping not only gives researchers an overview of the present state of knowledge, but also highlights fields that require further investigation and fields that are ready for a comprehensive synthesis. Meanwhile, bibliometrics helps researchers understand the relationships between scientific findings, illuminating the structure and development of a topic. This combinatorial approach called “scientometric analysis” provides researchers with a comprehensive overview of a particular area.

Though systematic reviews and meta-analyses are available in this field. Comprehensive, wide-scale analysis of trends and hotspots on this field are scarce, Thus, the purpose of this study is to give a comprehensive analysis of the publications on NMDAR’s role in depression over the last 20 years, highlight emerging trends and hotspots in the field, and serve as a guide for future research directions.Article types.

## 2 Materials and methods

### 2.1 Data source and search strategy

**FIGURE 1:**
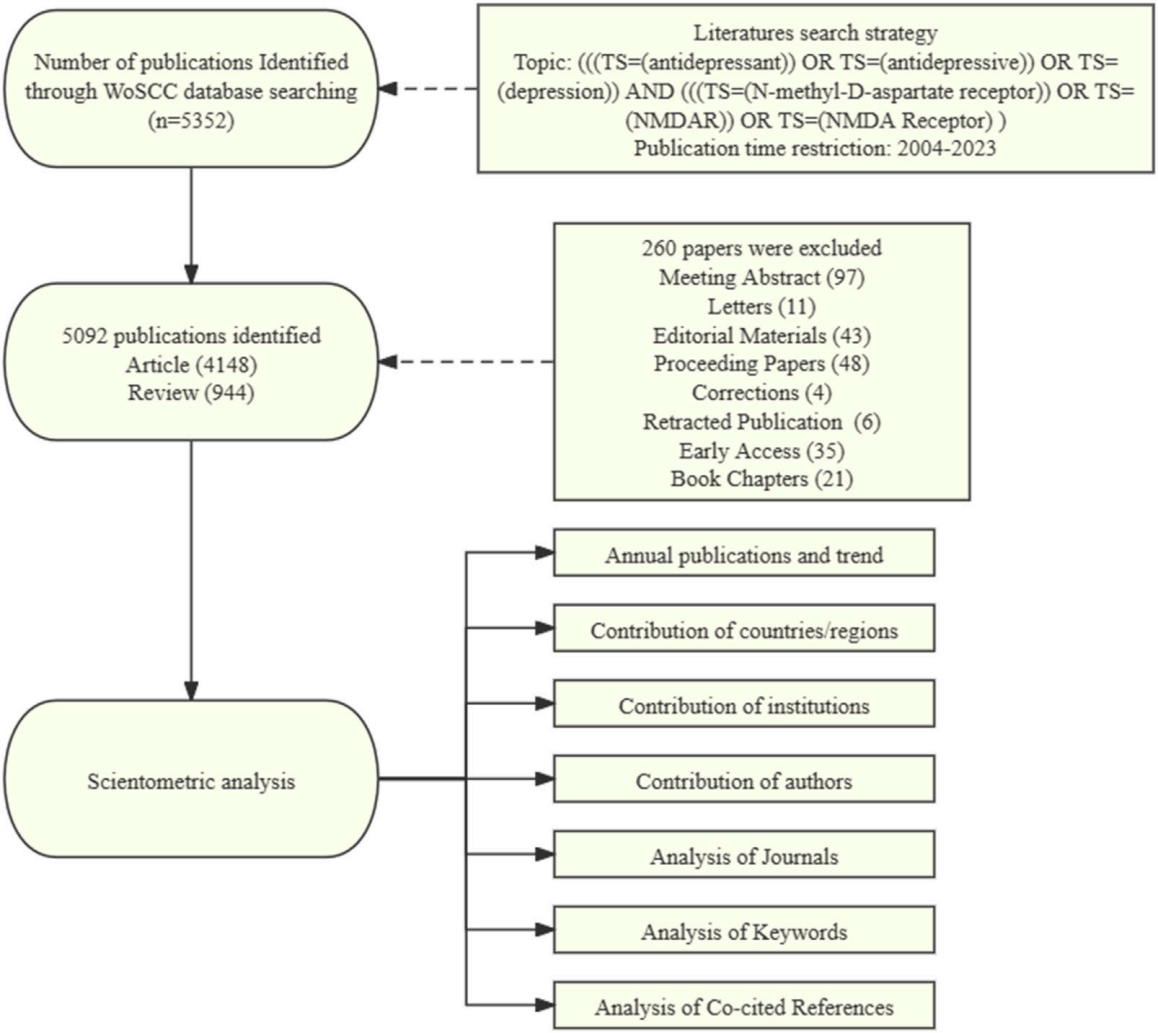
Flow chart of scientometric analysis.

In this study, we selected articles that were indexed in the Web of Science Core Collection (WoSCC) database, a sizable, multidisciplinary, worldwide, comprehensive, and highly influential academic database. Twenty thousand academic journals of the highest caliber and global influence, covering 250 subjects of study, are available on WoS. Our search formula was as follows: (((TS=(antidepressant)) OR TS=(antidepressive)) OR TS=(depression)) AND (((TS=(N-methyl-D-aspartate receptor)) OR TS=(NMDAR)) OR TS=(NMDA Receptor)), and we restricted the document type to “article” or “review”. There were no limitations on language. Publication date was from 2004/01/01 to 2023/11/12. All of the records and cited articles were exported as plain text files. The flowchart in Figure 1 illustrates the process of selecting publications.

### 2.2 Data extraction and analysis

To guarantee the validity of the findings, data extraction and literature screening were carried out by two separate researchers (Chen and Wang). From the obtained literature, we extracted and analyzed several elements such as the number of annual publications, countries or regions, institutions, authors, journals, keywords, and co-cited references.

The number of co-occurrences, citations and co-citations were the bibliometric metrics used in the analysis. The results of the systematic mapping included co-citation clusters, occurrence networks, citation networks, and co-citation networks.

CiteSpace was used to calculate metrics of significance, including betweenness centrality, silhouette score, modularity, citation bursts.

Betweenness centrality is a measure that describes the significance of a node in terms of the count of shortest paths through the node (Hansen et al., 2020). The higher the betweenness centrality of a node, the more important it is in the network. Nodes with betweenness centrality values ≥0.1 are defined as turning points and are highlighted with purple circles.

Modularity value (Q-value) is a metric of the strength of classifying networks into clusters or modules through data clustering approaches. Networks with high modularity include stronger connections among nodes inside the same cluster (Sabe et al., 2023). Weighted mean silhouette value (S-value) is a metric for assessing the effectiveness of the clustering algorithm, which calculates how similar a sample is to its own cluster in relation to other clusters. The Q-value >0.3 indicates a distinct significant clustering structure. The S-value is greater than 0.7 means the homogeneity between members within the cluster is good (Tao et al., 2022).

Utilizing the likelihood ratio method, cluster labels were extracted from the lists of co-cited references inside each cluster.

The R-Bibliometrix package tool was used to retrieve data about authors, nations/institutions, and journals. Author/country co-authorship network analysis and co-occurring keyword analysis were finished using VOSviewer (1.6.17). Collaboration network between institutions and co-cited reference analysis were preformed using Citespace (5.7.R5).

## 3 Results

### 3.1 Annual publications and trends

**FIGURE 2:**
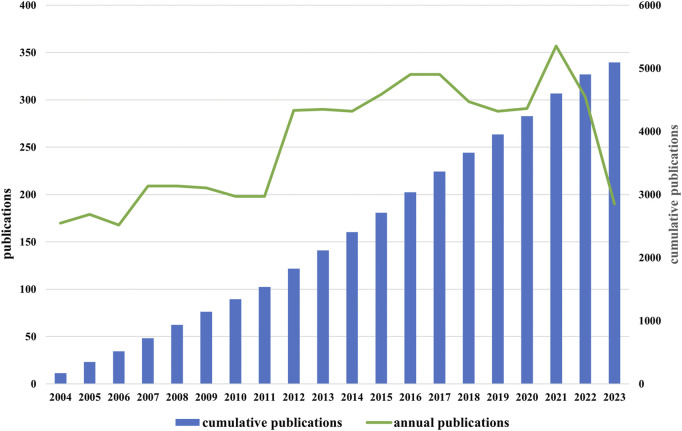
Trend of “NMADR in depression” publications from 2004 to 2023.

Through a search of WOSCC, 5,092 documents in total with the retrieval standards described above were included in this study, consisting of 4,148 articles (81.46%) and 944 reviews (18.53%). According to Figure 2, the number of annual publications in this area fluctuated since 2004, but overall it showed a increase trend. From 2004 to 2010, the number of publications fluctuated slowly, with an annual publication volume of less than 300. The volume of annual publications grew quickly between 2011 and 2017, surpassing 300 for the first time in 2015. Starting from 2018, the annual publication volume slightly decreased and maintained around 300 after reaching a peak of 357 articles in 2021. As of November 2023, the number of annual publications has slightly decreased compared to the previous year. Research on the role of NMDAR in depression has been growing and stabilizing after decades of investigation. This is indicative of the scientific community’s growing interest in this area.

### 3.2 Contributions of Countries or regions

**FIGURE 3:**
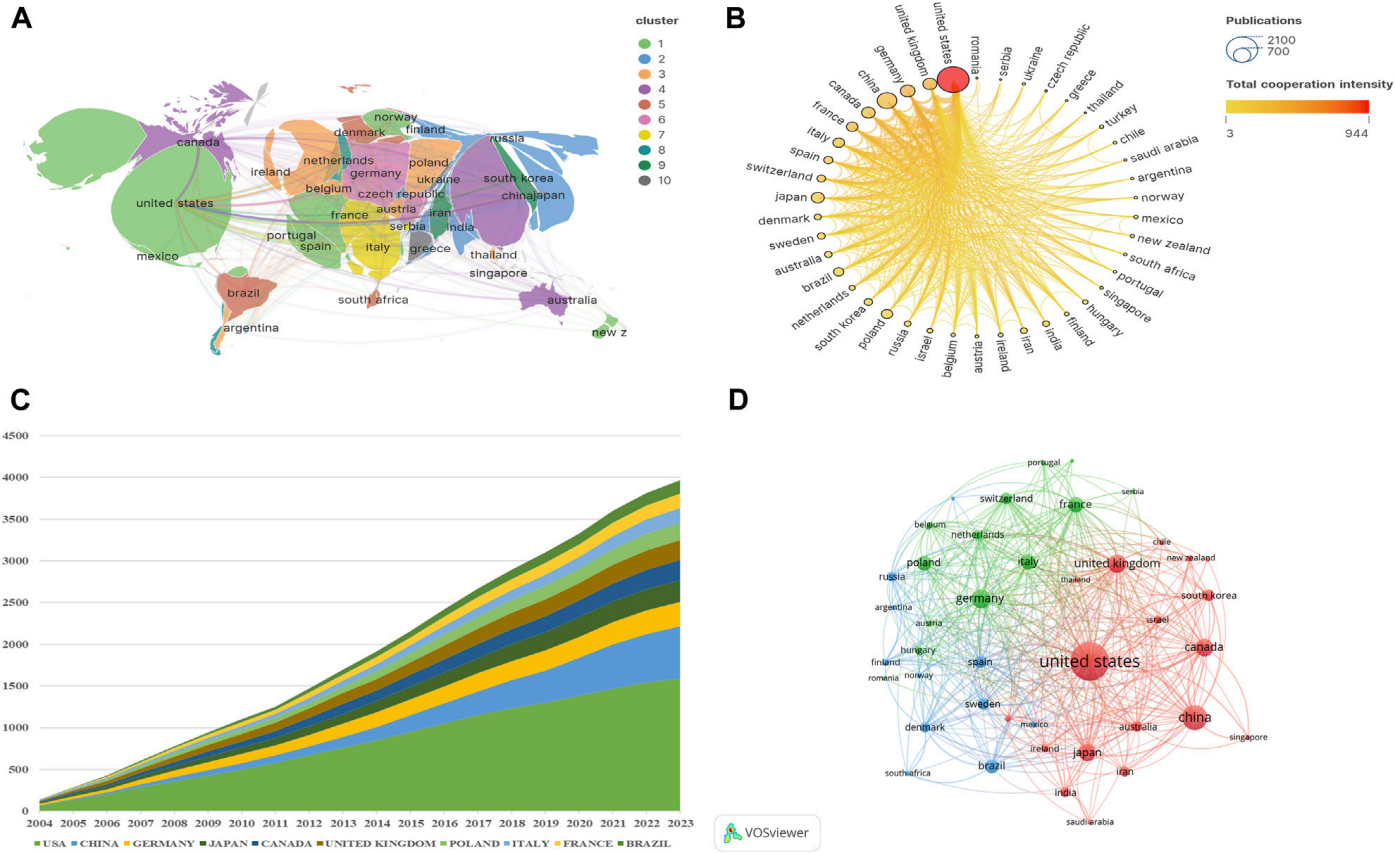
Contributions of Countries or Regions **(A)** Global distribution of publications. **(B)** Intensity of cooperation between countries. **(C)** Cumulative number of publications in the top 10 countries. **(D)** Country cooperation network. The size of the nodes represents the number of publications, and the thickness of the lines represents the frequency of cooperation between countries.

Since 2004, publications in this field had been published in a total of 72 countries (Figure 3A). Among them, there were 42 countries with a cumulative publication volume of over 8 articles. Table 1 displays the total number of publications (NP), total citations (TC), average citations (Avg.C), and H-index for the top ten countries producing the most work in the field of “NMDAR in Depression”. Among them, USA had the highest NP (1,590/31.2%), TC (124,277), Avg.C (78.20), and H-index (256), far surpassing other countries, followed by China (631/12.4%) and Germany (289/5.7%). China ranked second in terms of publication volume but had the lowest Avg.C (22.7), indicating that China needs to improve the impact of its research globally. There was a highly notable top effect and a highly unequal distribution of publications in this field among the nations. In this area, the United States leads research and collaborates closely with countries like China and the United Kingdom, (Figures 3B,D). Even though Switzerland, Denmark, and Sweden produced relatively little research, their lines’ strength and color showed that they had close ties to other countries and had a significant influence on the research of other countries. The growing interest and focus of nations on “NMDAR in Depression” is depicted in Figure 3C.

**TABLE 1:** The top 10 productive countries/regions.

Rank	Country	NP	TC	Avg.C	H-index
1	United States	1,590	124,277	78.20	256
2	China	631	14,346	22.70	89
3	Germany	289	12,128	42.00	96
4	Japan	263	9,907	37.70	84
5	Canada	238	10,558	44.40	93
6	United Kingdom	238	15,776	66.30	102
7	Poland	210	5,077	24.20	67
8	Italy	178	6,783	38.10	78
9	France	170	7,977	46.90	83
10	Brazil	160	5,533	34.60	66

### 3.3 Contributions of Institutions

**FIGURE 4:**
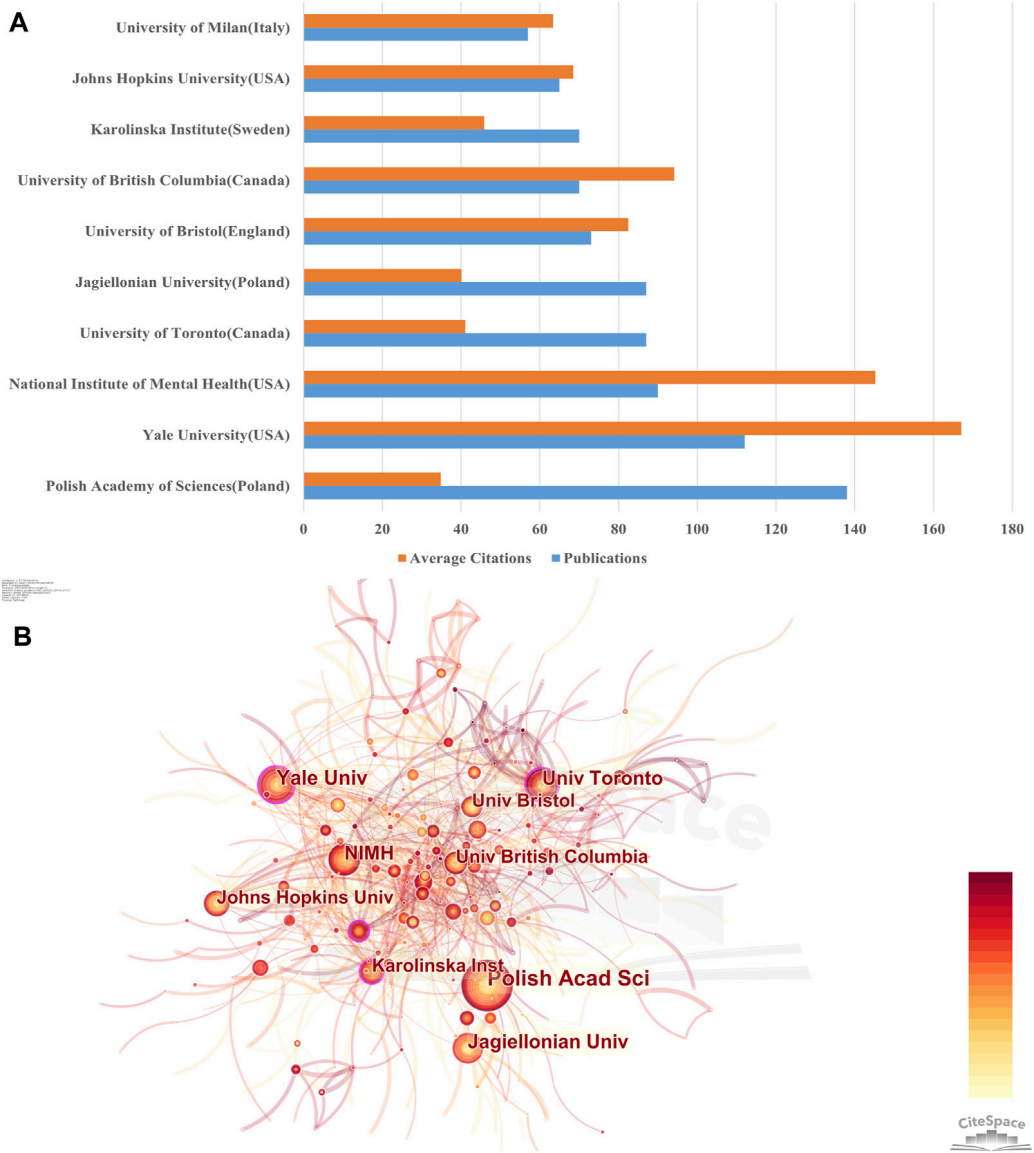
Contributions of Institutions **(A)** Total number of publications and average number of citations for the top 10 institutions in terms of publications. **(B)** Cooperation network of institutions. The size of the nodes represents the number of publications, and the thickness of the lines represents the frequency of cooperation between institutions. Nodes with centrality values ≥0.1 are defined as turning points and are highlighted with purple circles.

Publications on NMDAR in Depression were produced by more than 3,500 institutions. Using Citespace, co-authorship analysis of institutions was performed with a threshold of 30 documents, and 48 institutions were included in the analysis. According to Figure 4B, Polish Academy of Sciences (138) were notable leaders in the number of documents, followed by Yale University (112) and National Institute of Mental Health (90). National Institute of Mental Health, Yale University, and University of British Columbia, however, accounted for the top 3 with the most average citations and Polish Academy of Sciences Poland had the lowest average citations among the top ten institutions. As seen in Figure 4A, the centrality of Yale University, University of Toronto, and Karolinska Institute >0.1, indicating that these institutions maintain close connections with other organizations. It is worth noting that half of the universities or research institutes in the top 10 productivity rankings are in the United States or Poland. These outcomes demonstrate that they are at the forefront of the global.

### 3.4 Contributions of authors

**FIGURE 5:**
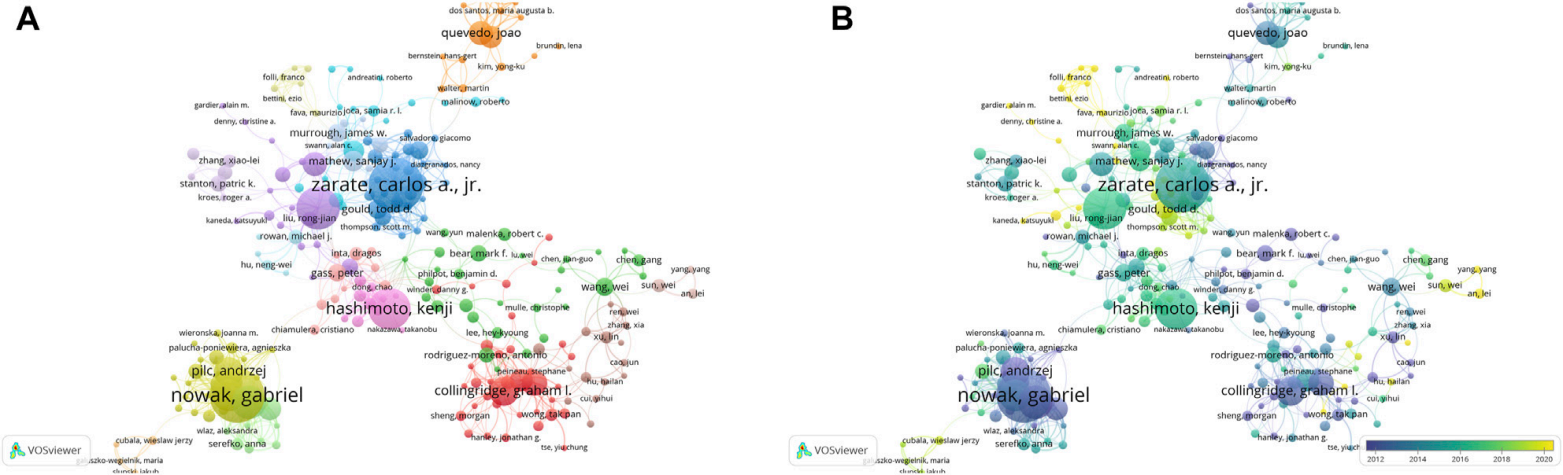
Authors co-authorship network maps by VOSviewer **(A)** Authors co-authorship network maps. **(B)** Overlay visualization of authors co-authorship network. The size of the nodes represents the number of publications, and the thickness of the lines represents the frequency of cooperation between authors. The nodes color indicates average publication year.

A totally 20,607 authors had contributed to the research on “NMDAR in Depression”. Zarate CA, Nowak G, and Rodrigues ALS were the three most productive authors in this field (Figure 5A; Table 2). Zarate CA, Duman RS and Hashimoto K, however, were more influential based on the H-index. Interestingly, Collingridge GL only published 34 publications, yet his average citations of 140 indicates that he had a significant impact in this field. Authors with more than six publications were included in the co-authorship network analysis. The authors were grouped into 17 clusters, as seen in Figure 5A. The largest cluster of co-authors was the red one, with 35 authors. Figure 5B demonstrates that Todd D. Gould, Panos Zanos and Kenji Hashimoto were emerging authors in the studies published in recent years, whereas the authors Andrzej Pilc, Ewa Poleszak and Gabriel Nowak were associated with the studies published in the earlier years.‬‬‬‬‬‬‬‬‬‬‬‬‬‬‬‬‬‬‬‬‬‬‬‬‬‬‬‬‬‬‬‬‬‬‬‬‬‬‬‬‬‬‬‬‬‬‬‬‬‬‬‬‬‬‬‬‬‬‬

**TABLE 2:** The top 10 productive authors.

Rank	Author	NP	TC	Avg.C	H-index	Start
1	Zarate CA	69	11,412	165.39	45	2006
2	Nowak G	60	2,560	42.67	33	2004
3	Rodrigues ALS	48	1925	40.10	28	2004
4	Duman RS	47	11,535	245.43	40	2008
5	Hashimoto K	47	3,548	75.49	33	2009
6	Poleszak E	39	1,443	37	24	2004
7	Szewczyk B	38	1,666	43.84	24	2004
8	Pilc A	37	2012	54.38	28	2004
9	WangY	36	941	26.14	16	2004
10	Collingridge GL	34	4,844	142.47	27	2004

### 3.5 Analysis of journals

Publications pertaining to “NMDAR in Depression” have been found in 768 journals overall. According to the H-index ranking, Table 3 displays the top ten influential Journals. With 288 articles published, Journal of Neuroscience has the most publications, followed by Neuropharmacology (198) and Neuroscience (135). Almost a quarter of the articles came from the top ten journals. The impact factor of the top 10 journals in 2022 ranged from 2.5 to 16.2. Based on 2023 JCR journal ranking, the majority of the top 10 influential journals were identified as Q1. Among them, Neuron has the highest impact factor and average number of citations. Yet, Journal of Neuroscience is another journal that has significantly influenced this field’s progress in terms of the H index.

**TABLE 3:** The top 10 influential journals in terms of h-index.

Rank	Journal	NP	TC	Avg.C	IF/JCR (2022)	H-index
1	Journal of Neuroscience	288	21,711	75.39	5.3 Q1	88
2	Neuron	69	13,002	188.43	16.2 Q1	57
3	Proceedings of the National Academy of Sciences of the United States of America	86	7,514	87.37	11.1 Q1	52
4	Neuropharmacology	198	9,146	46.19	4.7 Q2	46
5	Neuropsychopharmacology	102	8,384	82.20	7.6 Q1	46
6	Biological Psychiatry	60	7,478	124.63	10.6 Q1	42
7	Molecular Psychiatry	64	5,903	92.23	11.0 Q1	42
8	Journal of Neurophysiology	95	3,061	32.22	2.5 Q3	34
9	Journal of Physiology-London	65	2,812	43.26	5.5 Q1	34
10	Neuroscience	135	4,049	30.00	3.3 Q3	34

### 3.6 Analysis of keywords

**FIGURE 6:**
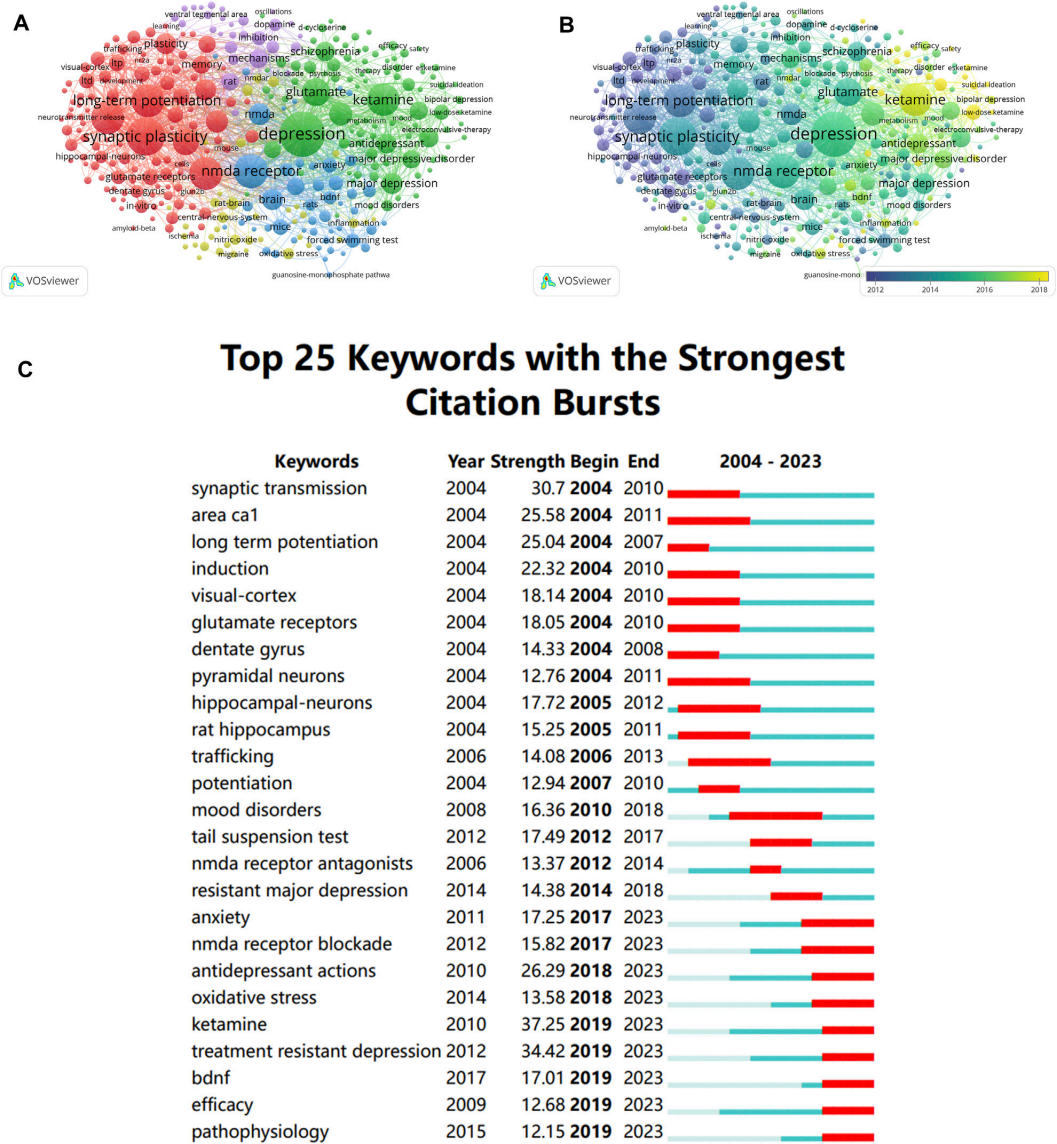
Analysis of keyword **(A)** Keywords co-occurrence network map. **(B)** Overlay visualization of keywords co-occurrence network. The size of the nodes represents the number of keyword occurrences, and the nodes color indicates average publication year. **(C)** The top 25 keywords of “NMADR in depression” with the highest citation bursts.

As the core for a paper, keywords may indicate research trends and hotspots within a topic when they are analyzed for co-occurrence. Among all the keywords retrieved from the articles, 321 were discovered to have more than 30 occurrences. As is shown Figure 6A, each keyword represents a node, and the larger the node, the more times the keyword appears, and the more it can represent the hotspots in this field. Besides, the same-colored nodes in Figure 6A indicate that these keywords belong to the same cluster, and there were five clusters altogether. “synaptic plasticity,” “glutamate receptors,” and “long term depression,” make the majority of the red cluster. The green cluster is primarily concerned with the classification and treatment of depression. The purple cluster is mainly associated with the mechanisms of antidepressants. And the blue one is associated with animal experiment and NMDAR. Additionally, the overlay visualization map displayed the average year of publication for each keyword that appeared in these articles, and it revealed that keywords of “NMDAR in Depression” were gradually shifting from the structure and function of NMDAR to NMDAR antagonists and antidepressants. (Figure 6B).

Burst keyword is the occurrence of a term appearing frequently during a specific period of time. Thus, not only can we identify how research hotspots have changed over time, but we can also examine current research trends and offer recommendations for further research. CiteSpace software was utilized to analysis the top 25 keywords with the strongest citation bursts. As shown in Figure 6C, we can see that early research hotspots were mostly found in synaptic transmission, long-term potentiation induction, and synaptic plasticity. However, “Anxiety”, “NMDA receptor blockade”, “Antidepressant”, “Oxidative stress”, “Treatment resistant depression”, “Ketamine” and “BDNF” have been the keywords with the strongest citation bursts in recent years. Based on the results mentioned above, it is reasonable to assume that research hotspots and future trends in this field will be strongly associated with the treatment of MDD and TRD. In addition, mechanisms and targets of NMDAR antagonists are a focus of current and future research.

### 3.7 Analysis of Co-cited references

When two or more publications are cited simultaneously by one or more subsequent papers, this is referred to as a co citation relationship. The top 10 co-cited papers in “NMDAR in Depression” are displayed in Table 4. The most co-cited publication was authored by Berman RM on Biological Psychiatry in 2000, who initially discovered that ketamine (an NMDAR antagonist) showed a rapid antidepressant effect (Berman et al., 2000). Then, Zarate CA et al. authored the publication with the second most co-citations, and they conducted another trial on patients with TRD. As a result, they found the robust and rapid antidepressant effects of NMDAR antagonist (Zarate et al., 2006). Furthermore, it is worth mentioning that Zarate CA’s subsequent research published in Science in 2010 discovered that mTOR-dependent synapse formation contributes to the quick antidepressant effects of NMDA antagonists (Li et al., 2010).

**TABLE 4:** The top 10 co-cited references.

Rank	Title	First author	Year	Journal (IF/JCR)	Co-citations
1	Antidepressant effects of ketamine in depressed patients	Berman, RM	2000	BIOLOGICAL PSYCHIATRY (10.6/Q1)	830
2	A randomized trial of an N-methyl-D-aspartate antagonist in treatment-resistant major depression	Zarate, CA	2006	ARCHIVES OF GENERAL PSYCHIATRY (14.4/Q1)	791
3	mTOR-Dependent Synapse Formation Underlies the Rapid Antidepressant Effects of NMDA Antagonists	Li, NX	2010	SCIENCE (56.9/Q1)	656
4	NMDA receptor blockade at rest triggers rapid behavioural antidepressant responses	Autry, AE	2011	NATURE (64.8/Q1)	532
5	Cellular mechanisms underlying the antidepressant effects of ketamine: Role of α-amino-3-hydroxy-5-methylisoxazole-4-propionic acid receptors	Maeng, S	2008	BIOLOGICAL PSYCHIATRY (10.6/Q1)	428
6	LTP and LTD: An embarrassment of riches	Malenka, RC	2004	NEURON (16.2/Q1)	360
7	A synaptic model of memory: long-term potentiation in the hippocampus	Bliss, TV	1993	NATURE (64.8/Q1)	332
8	Glutamate N-methyl-D-aspartate Receptor Antagonists Rapidly Reverse Behavioral and Synaptic Deficits Caused by Chronic Stress Exposure	Li, NX	2011	BIOLOGICAL PSYCHIATRY (10.6/Q1)	327
9	NMDAR inhibition-independent antidepressant actions of ketamine metabolites	Zanos, P	2016	NATURE (64.8/Q1)	323
10	Activation of glutamatergic neurotransmission by ketamine: a novel step in the pathway from NMDA receptor blockade to dopaminergic and cognitive disruptions associated with the prefrontal cortex	Moghaddam, B	1997	JOURNAL OF NEUROSCIENCE (5.3/Q1)	313

**FIGURE 7:**
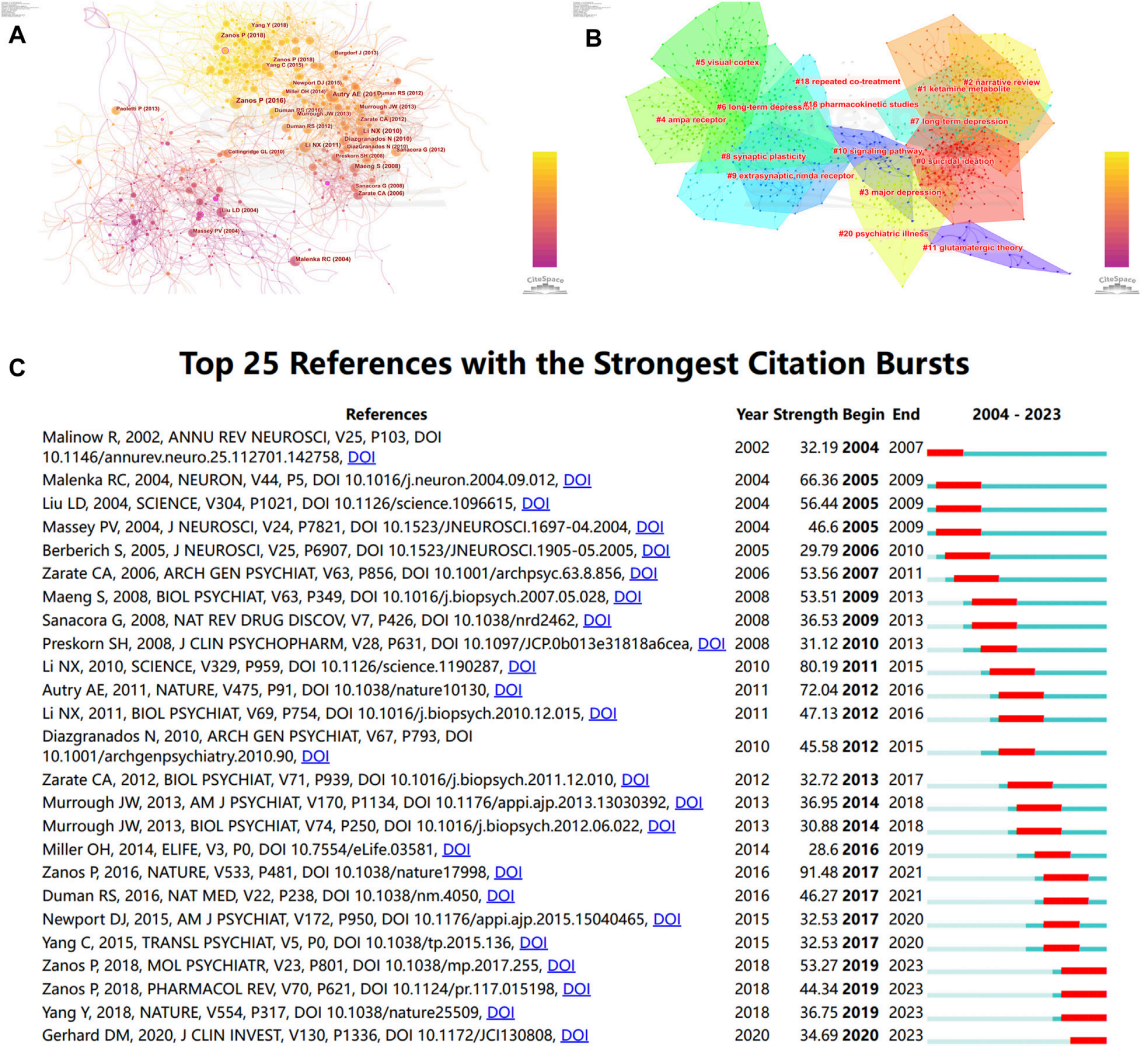
Analysis of co-cited references by CiteSpace **(A)** Co-cited references network visualization **(B)** and its corresponding clusters. The calculated clusters are displayed on this network. In this network, every node represents an individual publication, and the size of a node is directly correlated with the number of co-citations of that publication. **(C)** The top 25 references of “NMADR in depression” with the highest citation bursts.

As is shown in Figure 7A, we created a co-cited reference network utilizing the CiteSpace software, then clustered them and showed cluster label by log-likelihood ratio (Figure 7B). 15 highly credible main clusters were identified with notable modularity (Q-value = 0.7628) and silhouette scores (S-value = 0.9171). Cluster#0 (“suicidal ideation”) is the largest cluster that has “bipolar depression”, “antidepressant efficacy”, “randomized controlled trial”, “treatment-resistant major depression”, and “neurocognitive effect” as the top keywords. Cluster#1 (“ketamine metabolite”) was the second largest and had “prototype antidepressant”, “potential mechanism”, “gamma power”, “long-term depression”, and “crossover trial” as the keywords. These findings suggest that the current focus is on Ketamine and its metabolites, potential mechanism, treatment-resistant major depression, synaptogenesis, and NMDA receptor antagonists. At last, we screened out the top 25 references with the strongest citation bursts (Figure 6C), indicating that these papers have been frequently cited in a short period of time. It is obvious that “Zanos P, 2016, NATURE, V533, P481” (Zanos et al., 2016) had the highest citation burst strength of 94.81. Furthermore, from 2018 to 2023, “Zanos P, 2018, MOL PSYCHIATR, V23, P801” (Zanos and Gould, 2018) and “Zanos P, 2018, PHARMACOL REV, V70, P621” (Zanos et al., 2018) had the highest citation burst strengths, suggesting that Zanos P has been actively conducting research in this area lately and giving us clues about potential hotspots for future study in this area.

## 4 Discussion

### 4.1 Main findings

In this study, a scientometric analysis was conducted to better elucidate current research trends regarding the role of NMDA receptor in the treatment of depression. Scientometric analyses, in contrast to systematic reviews, are able to forecast future directions of study in a specific field by effectively synthesizing findings that have been published so far. Annual publications can provide a general overview of the development process of NMDAR in the treatment of depression. We observed a steady increase in the number of annual publications in this field. This suggests that depression is still a field of great interest, and that NMDAR is an important therapeutic target for depression. Therefore, future research may continue to emphasize NMDAR in the treatment of depression.

Countries, institutions, and authors’ levels of scientific research are judged in large part by the number and quality of publications they produce in a particular field. Among countries, USA had the highest number of publications, total citations, average citations, and H-index, far surpassing other countries. This phenomenon demonstrates that the United States has made the greatest contribution and is in a leadership position in this field. Even though Switzerland, Denmark, and Sweden produced relatively little research, they had close ties to other countries and had a significant influence on the research of other countries. Research of NMDAR in the treatment of depression has received extensive attention worldwide, and there is a need to further strengthen international cooperation in the future. Polish Academy of Sciences were notable leaders in the number of documents. However, out of the top ten institutions, the Yale University has the highest average citations, indicating that it is the most influential institution across the world.

Zarate CA and Nowak G were the two most productive authors in this field. Zarate CA and Duman RS, however, were more influential based on H-index and citations. In addition, Berman has also made significant contributions to the field. His most frequently cited publication was published in 2000 under the title “Antidepressant effects of ketamine in depressed patients” (Berman et al., 2000). This clinical trial is the first to report the rapid antidepressant effect of ketamine. After that, Zarate CA carried out a double-blind randomized controlled trial, and the results showed that ketamine was effective in treating patients with resistant major depression who had not responded to at least two traditional antidepressant treatments (Zarate et al., 2006). Duman RS has led or participated in numerous studies on NMDAR. One of Duman’s highly cited papers was published in Science in 2010 (Li et al., 2010). In this study, they found that ketamine rapidly activated the mammalian target of rapamycin (mTOR) signaling, increasing the number and functionality of new spine synapses in rats as well as synaptic signaling proteins in the prefrontal cortex. The rapid antidepressant action of ketamine may be facilitated by these effects.

As for journals, Journal of Neuroscience is a prestigious journal with the largest number of articles published in the field. Authors may consider this journal for their manuscripts. Among the top 10 journals, the majority of them were identified as Q1. Consequently, research quality and article quality are very high in this field of study. Furthermore, scholarly journals possessing elevated JCR rankings and impact factors can function as superior references for our work.

Notably, as Figure 5A illustrates, a bibliometric analysis of keywords revealed the existence of several publication clusters. For instance, “synaptic plasticity”, “classification and treatment of depression”, “mechanism of antidepressant”, and “animal experiment”. In addition, the overlay visualization map revealed that NMDAR antagonists and antidepressants are emerging keywords in recent years (Figure 6B). Figure 6C illustrates that the majority of early research hotspots were related to synaptic plasticity and synaptic transmission. Nevertheless, the keywords with the highest citation bursts in recent years have been “NMDA receptor blockade”, “Antidepressant”, “Oxidative stress”, “Treatment resistant depression”, “Ketamine”, “Pathophysiology”, and “BDNF”. In summary, these results raise significant concerns about the correlation between NMDAR antagonists and depression.

### 4.2 NMDAR antagonists

NMDAR antagonists are gaining more and more interest as a class of medications with potential treatment benefits for a wide range of disorders. NMDAR antagonists influence glutamatergic signaling by fully or partially blocking NMDAR. Four binding sites were identified in the receptor: one on the N-terminal domain (NTD), one on the glutamate or glycine binding sites, and one on the ion channel pore (Monaghan et al., 2012). NMDARs’ complicated structure allows for various sites of inhibition, which makes them potential targets for a variety of drugs. NMDAR antagonists can be competitive or noncompetitive. There are two types of uncompetitive NMDA receptor blockers: partially trapping blockers and trapping blockers (Szewczyk et al., 2012). When taken properly, NMDAR antagonists have a positive safety profile despite long-standing concerns about their side effects. Additionally, they are a promising family of medications that may produce effective therapies for a variety of CNS illnesses due to their rapid-acting mechanism of action, which produces rapid effects in comparison to other therapeutic drugs. In the past two decades, research has mainly focused on NMDAR antagonists for the treatment of depression. Numerous clinical and preclinical research has demonstrated the rapid antidepressant properties of NMDAR antagonists (Berman et al., 2000; Zarate et al., 2006; Amidfar et al., 2018a; Amidfar et al., 2018b; Fava et al., 2020; Dwyer et al., 2021; Tabuteau et al., 2022).

### 4.3 Ketamine

The first NMDAR antagonist to be utilized in clinical practice, ketamine was demonstrated to have fast antidepressant effects in MDD patients (Berman et al., 2000). The safety and effectiveness of ketamine and esketamine for the treatment of major depressive disorders have been confirmed by further research. Many randomized controlled trials have demonstrated that intravenous sub-anesthetic doses of ketamine (0.5 mg/kg over 40 min) produce rapid and sustained antidepressant actions (Nikolin et al., 2023). In addition, ketamine has demonstrated efficacy in treating TRD (Phillips et al., 2019) and bipolar disorder (Diazgranados et al., 2010). The S-enantiomer of ketamine, known as esketamine, has been developed as an intranasal antidepressant. Randomized placebo-controlled trials have shown that esketamine is safe and effective in long-term use (Daly et al., 2018; Popova et al., 2019). Furthermore, for MDD patients who are at immediate risk of suicide, intranasal esketamine quickly reduces depressed symptoms like suicidal thoughts (Canuso et al., 2018). Likewise, ketamine was shown in numerous studies to rapidly ameliorate suicidal ideation and the threat of suicide in TRD patients (Abbar et al., 2022; Grunebaum et al., 2018; Ionescu et al., 2019; Ionescu et al., 2016; Wilkinson et al., 2018). Despite the rapid and robust antidepressant effects of ketamine and other NMDAR antagonists, their abuse potential, dissociative side effects, and neurotoxicity limit their use (Short et al., 2018). Furthermore, there is a higher chance of severe psychosis and euphoria with repeated use (Machado-Vieira et al., 2009). Further study on NMDAR antagonists, in particular selective NMDAR subunit antagonists, is necessary since NMDAR seem to be potential target for developing more effective and safer therapeutic strategies.

### 4.4 Mechanisms of ketamine

Currently, ketamine is mainly used in research due to its adverse reactions. However, by comprehending the molecular mechanisms underlying its therapeutic effects, we may be able to identify new targets for novel rapid-acting antidepressants. The mechanism of action of NMDAR antagonists as rapid antidepressants mainly includes synaptic plasticity and NMDAR blockade (blockade of spontaneous NMDAR-mediated transmission, blockade of NMDAR on GABAergic inhibitory interneurons, and blockade of extra-synaptic NMDAR).

Duman et al. suggest that rapid alterations in synaptic transmission and function are the molecular and cellular mechanisms responsible for the rapid antidepressant effects of ketamine and other NMDAR antagonists (Duman et al., 2016). Synaptic plasticity signaling systems including BDNF signaling (Lepack et al., 2015; Amidfar et al., 2018a; Zanos and Gould, 2018), mTOR signaling (Li et al., 2010; Workman et al., 2013), eukaryotic elongation factor 2 kinase (eEF2K) (Autry et al., 2011) and glycogen synthase kinase-3 (GSK-3) (Beurel et al., 2011; Liu et al., 2013) are engaged in mediating the antidepressant effects of NMDAR antagonists. In addition, Monteggia and colleagues recently discovered that the antidepressant effects of ketamine were significantly reliant on a novel type of rapid homeostatic synaptic plasticity (Kavalali and Monteggia, 2020). Synapse-specific changes in synaptic strength caused by Hebbian types of plasticity follow the direction of the initial conditioned stimulus (Kavalali and Monteggia, 2023). However, homeostatic plasticity counteracts substantial increases or decreases in activity by rebalancing synaptic strength with negative feedback. Due to the possibility of little disruption to cognitive function, this characteristic of homeostatic synaptic scaling makes it the desirable kind of plasticity for mediating ketamine actions.

According to studies conducted in the Duman lab, ketamine acts via blocking NMDARs on GABAergic interneurons (Gerhard et al., 2020; Pothula et al., 2021). It is hypothesized that this brief decrease in excitatory drive to these NMDARs expressed on inhibitory GABAergic interneurons will lower GABA release tonic release and disinhibit target excitatory neuron activity. The subsequent rise in glutamatergic activity causes the function of the mammalian target of rapamycin complex 1 (mTORC1) to be activated downstream, increasing the development of dendritic spines and synaptic protein synthesis, therefore producing the rapid and sustained antidepressant actions (Li et al., 2010; Workman et al., 2013; Moda-Sava et al., 2019). On the other hand, ketamine and other NMDAR antagonists have been demonstrated to inhibit NMDAR-mediated neurotransmission at rest. This results in a de-suppression of protein synthesis, which in turn causes synaptic potentiation in the hippocampal CA1 area and behavioral effects (Autry et al., 2011; Nosyreva et al., 2013). It is hypothesized that the antidepressant effects of ketamine are partly intermediated by spontaneous NMDAR-mediated neurotransmission, which increases synaptic neurotransmission via a protein synthesis-dependent process including BDNF and eEF2K. Another hypothesis proposes that ketamine selectively inhibits extra-synaptic GluN2B-containing NMDARs, therefore blocking ambient glutamate-driven tonic activation of these receptors and further inducing excitation in pyramidal neurons (Miller et al., 2014). Blockade of extra-synaptic NMDARs leads to de-suppression of mTOR function, which in turn promotes protein synthesis and antidepressant effects.

Monteggia lab explored the mechanism of ketamine’s sustained antidepressant actions (Kim et al., 2021), and they found that the translation-dependent effects of ketamine are essential for initiating its sustained actions. Initial synaptic plasticity elicits transcriptional modulation, which is required for the sustained effects. Furthermore, the particular function of Methyl-CpG-binding protein 2 (MeCP2) is necessary for the transcriptional modulation of sustained ketamine actions. According to a recent study by Hu and colleagues published on Science, ketamine can block NMDARs in the lateral habenula (LHb) and reduce burst firing for up to 24 h following a single systemic injection. The use-dependent trapping of ketamine in NMDARs is necessary for the long-term blockage of NMDARs, resulting in sustained antidepressant effects.

Overall, three sequential processes are assumed to be responsible for the rapid antidepressant effects of ketamine. First, blockade of NMDAR expressed on inhibitory interneurons causes derepression of presynaptic neurons, which in turn causes an influx of glutamate into the synaptic gap. Then, there is an enhanced activation of AMPAR together with extra-synaptic NMDAR blockade by ketamine. eventually, these general changes in glutamatergic transmission initiate signaling systems associated with postsynaptic neuroplasticity (Driver et al., 2022). In addition, activation of AMPAR may modulate signaling pathways involved in synaptic plasticity, including promoting the release of BDNF and triggering the activation of tropomyosin receptor kinase B (TrkB) and mTORC1 (Yavi et al., 2022).

## 5 Strengths and limitations

Through the application of bibliometrics and systematic mapping, a scientometrics approach is proposed. Scientometric analyses provide a comprehensive overview of the field of study which cannot be acquired from systematic reviews or meta-analyses alone. The networks derived from this kind of analysis visualize areas of research focus as well as associations between authors, countries, and institutions. Researchers can utilize this information in a variety of ways to guide current and future research and clinical practice. However, there were also some limitations to this paper. Firstly, we only retrieved data from the WoSCC database, and publications not included in the WoSCC database were omitted. Future studies could expand the inclusion of literature, merge databases, and conduct updated scientometric analysis to ensure completeness. Second, because the co-citation analysis focuses only on the first author, it may not fully capture the impact of all authors. Finally, it is possible that most cutting-edge, novel studies, especially from smaller institutions are disregarded. Regarding the scientometric analysis, two possible sources of bias are publication bias and bias against novelty. There is a bias against innovation in research since novel publications may be published in journals with lower impact factors, have larger citation variance, and take longer to be recognized (Wang et al., 2017). Publication bias occurs when authors, editors, and reviewers choose to publish research with significant results over those with non-significant ones for a variety of reasons, including lack of interest or contradictory results that do not fit into planned goals (DeVito and Goldacre, 2019). As a result, potentially influential publications may not have been included in our analysis due to low citation counts.

## 6 Conclusion

As the first scientometric analysis of NMDAR in Depression, this study sheds light on the development, trends, and hotspots of research about NMDAR in Depression worldwide, which may be useful to other researchers. The mechanisms of ketamine and other NMDAR antagonist, synaptic plasticity and neuroplasticity related downstream factors in antidepressant action of NMDAR antagonists are hot research topics at present. The adverse effects of NMDAR antagonist like ketamine and the risk of neurotoxicity associated with long-term use have prompted research on new rapid acting antidepressants.

Further study of the mechanism of action of NMDAR antagonists will contribute to the development of novel rapid-acting antidepressants especially for TRD patients. In addition, large-scale clinical trials are necessary to assess the antidepressant efficacy and safety of other oral NMDAR antagonists. NMDAR modulators, such as subunit-selective NMDAR antagonist, NMDAR partial agonists, and positive allosteric modulators of NMDAR require more attention because they may be future perspective antidepressants. These studies will further advance our understanding of depression and will contribute to the development of safer and more effective rapid-acting antidepressants.

## Data Availability

The original contributions presented in the study are included in the article/Supplementary material, further inquiries can be directed to the corresponding authors.
